# Health-related quality of life impact of cobimetinib in combination with vemurafenib in patients with advanced or metastatic *BRAF*^V600^ mutation–positive melanoma

**DOI:** 10.1038/bjc.2017.488

**Published:** 2018-02-13

**Authors:** Brigitte Dréno, Paolo A Ascierto, Victoria Atkinson, Gabriella Liszkay, Michele Maio, Mario Mandalà, Lev Demidov, Daniil Stroyakovskiy, Luc Thomas, Luis de la Cruz-Merino, Caroline Dutriaux, Claus Garbe, Karen Bartley, Thomas Karagiannis, Ilsung Chang, Isabelle Rooney, Daniel O Koralek, James Larkin, Grant A McArthur, Antoni Ribas

**Affiliations:** 1 Department of Dermato Cancerology, Nantes University, Nantes 44093, France; 2 Istituto Nazionale Tumori Fondazione G. Pascale, Naples 80131, Italy; 3 Princess Alexandra Hospital, Woolloongabba, QLD 4102, Australia; 4 National Institute of Oncology, Budapest 1122, Hungary; 5 Azienda Ospedaliera Universitaria Senese, Siena 53100, Italy; 6 Department of Oncology and Haematology, Papa Giovanni XXIII Hospital, Bergamo 24127, Italy; 7 N. N. Blokhin Russian Cancer Research Center, Moscow 115478, Russia; 8 Moscow City Oncology Hospital 62, Krasnogorsk 14301, Russia; 9 Service de Dermatologie, Centre Hospitalier Lyon Sud, Pierre-Bénite 69495, France; 10 Hospital Universitario Virgen Macarena, Seville 41009, Spain; 11 Hôpital Saint André, Bordeaux 33075, France; 12 Department of Dermatology, University of Tübingen, Tübingen 72074, Germany; 13 Genentech, Inc., South San Francisco, CA 94080, USA; 14 The Royal Marsden NHS Foundation Trust, London SW3 6JJ, UK; 15 Peter MacCallum Cancer Centre, East Melbourne, VIC 3002, Australia; 16 University of Melbourne, Parkville, VIC 3052, Australia; 17 Jonsson Comprehensive Cancer Center, University of California Los Angeles, Los Angeles, CA 90095, USA

**Keywords:** vemurafenib, cobimetinib, MEK inhibitor, BRAF inhibitor, HRQOL, EORTC QLQ-C30, metastatic melanoma

## Abstract

**Background:**

In the coBRIM study, cobimetinib plus vemurafenib (C+V) significantly improved survival outcomes *vs* placebo and vemurafenib (P+V) in patients with advanced/metastatic *BRAF*^V600^-mutated melanoma. An analysis of health-related quality of life (HRQOL) from coBRIM is reported.

**Methods:**

Patients completing the European Organisation for Research and Treatment of Cancer Quality of Life Questionnaire Core 30 (QLQ-C30) at baseline and ⩾1 time point thereafter constituted the analysis population. Change from baseline ⩾10 points was considered clinically meaningful.

**Results:**

Mean baseline scores for all QLQ-C30 domains were similar between arms. Most on-treatment scores for QLQ-C30 domains were also comparable between arms. A transient deterioration in role function in cycle 1 day 15 (C1D15; -14.7 points) in the P+V arm and improvement in insomnia in the C+V arm at C2D15 (-12.4 points) was observed. Among patients who experienced a ⩾10-point change from baseline (responders), between-group differences were greatest for insomnia (16%), social functioning (10%), fatigue (9%) and pain (7%), all favouring C+V. Diarrhoea, photosensitivity reaction, pyrexia, and rash did not meaningfully affect global health status (GHS). Serous retinopathy was associated with a transient decrease in GHS at C1D15 assessment.

**Conclusions:**

In patients with advanced/metastatic *BRAF*^V600^-mutated melanoma, treatment with C+V maintained HRQOL compared with P+V, with superior efficacy.

The combination of cobimetinib (a MEK inhibitor) and vemurafenib (a BRAF inhibitor) has been shown to significantly improve clinical outcomes in patients with *BRAF*^V600^ mutation–positive unresectable locally advanced or metastatic melanoma. In the phase III coBRIM study, cobimetinib combined with vemurafenib significantly improved investigator-assessed progression-free survival (PFS) compared with placebo and vemurafenib in this patient population (median PFS, 9.9 *vs* 6.2 months; hazard ratio=0.51; 95% confidence interval (CI): 0.39–0.68; *P*<0.001 (data cutoff May 2014; database lock 10 July 2014)) (Larkin *et al*, 2014).

In addition to efficacy benefits, both tolerability of treatment and health-related quality of life (HRQOL) are important considerations for patients. Accordingly, we evaluated patient-reported outcomes (PROs), including symptoms, functional impact, and HRQOL, in patients participating in the coBRIM study using the European Organisation for Research and Cancer Quality of Life Questionnaire Core 30 (EORTC QLQ-C30; Aaronson *et al*, 1993). The EORTC QLQ-C30, a validated, self-reported measure, consists of 30 items which assess five functional domains (physical, role, cognitive, emotional, and social), global health status (GHS), and various general cancer symptoms.

This report is based on the data cutoff of the aforementioned primary PFS analysis (May 2014). At the time of the data cutoff, cobimetinib combined with vemurafenib was associated with a similar incidence of any-grade adverse events (AEs) compared with placebo and vemurafenib (98% of patients in each arm). The rate of treatment discontinuation because of AEs was also similar in both arms (13% in the cobimetinib combined with vemurafenib arm and 12% in the placebo and vemurafenib arm) (Larkin *et al*, 2014). However, certain AEs, including diarrhoea, photosensitivity, pyrexia, rash, and serous retinopathy, occurred more frequently with this combination regimen, and were of clinical interest. Therefore, the objectives of these analyses were twofold: to evaluate the impact of treatment with cobimetinib combined with vemurafenib on patient HRQOL compared with placebo and vemurafenib, and to explore the impact of select symptomatic AEs on HRQOL in the treatment arm.

## Materials and methods

### Study design and treatment

The coBRIM study (NCT01689519) was a global multicentre, randomised, double-blind, placebo-controlled phase III trial designed to evaluate the safety and efficacy of cobimetinib in combination with vemurafenib, compared with placebo and vemurafenib, in patients with *BRAF*^V600^ mutation–positive unresectable locally advanced or metastatic melanoma. Patient eligibility criteria have previously been described (Larkin *et al*, 2014). Key eligibility criteria included age ≥18 years, histologically confirmed unresectable locally advanced stage IIIC or IV melanoma, *BRAF*^V600^ mutation detected using the cobas^®^ 4800 BRAF V600 Mutation Test (Roche Molecular Systems Inc, Indianapolis, IN, USA), no prior systemic therapy for advanced disease, and Eastern Cooperative Oncology Group performance status (ECOG PS) 0 or 1.

Patients were randomly assigned in a 1:1 ratio to receive oral vemurafenib (960 mg twice daily) in combination with either placebo or oral cobimetinib (60 mg once daily for 21 days followed by 7 days off; 21/7). Treatment was administered in 28-day cycles and continued until disease progression, unacceptable toxicity, or withdrawal of consent. A prespecified secondary end point was to compare HRQOL between treatment arms, as measured by the GHS scale within the EORTC QLQ-C30. The study was approved by the institutional review board or ethics committee at each participating institution and was conducted in accordance with the provisions of the Declaration of Helsinki and the International Conference on Harmonisation guidelines for Good Clinical Practice. All patients provided written informed consent.

### HRQOL assessments

Disease and treatment-related symptoms, functioning, and HRQOL were evaluated using the EORTC QLQ-C30 (Aaronson *et al*, 1993). Functional domains and symptom-related items are measured over the previous week on a 4-point scale, ranging from ‘not at all’ to ‘very much’. The GHS 2-item scale is intended to measure HRQOL and requests patients to select the number (1–7) that best applies to them in terms of their overall health as well as their ‘overall quality of life’ during the past week. For GHS and functioning scales, an increase in scores indicates improvement, whereas a decrease in scores indicates improvement for symptom scales or items.

The EORTC QLQ-C30 was administered at baseline, days 1 and 15 in cycles 1 and 2, and every second cycle thereafter until patient withdrawal or end of study. Questionnaires were collected using electronic tablets and data were transmitted directly into the study database. Missing values were not imputed. Data were evaluable through cycle 8 day 1, after which too few patients remained to allow for meaningful conclusions (<25% of the baseline cohort). Change of ⩾10 points from baseline in any scale (GHS, functioning, or symptoms) was considered clinically meaningful (Osoba *et al*, 1998).

### Statistical analysis

Patients who completed a PRO assessment at baseline and at least one time point after baseline were defined as the PRO-evaluable population and were included in the analysis. The PRO end points were not part of formal statistical testing and the study was not powered to detect differences between treatment arms.

We reported descriptively the mean change from baseline scores for each EORTC QLQ-C30 scale at each assessment time point by treatment arm. Additionally, repeated-measures mixed-effects models were also constructed for the comparison of the EORTC QLQ-C30 GHS, function, and symptom scores between treatment arms, to provide a summary statistical measure of longitudinal change. Each model had a term for intercept, a term for a linear time trend (in weeks), a term for treatment group, a term for treatment-by-time interaction, and stratification factors, including geographic region and metastasis classification. Repeated measures over time were accounted for by an exchangeable covariance structure (using the REPEATED statement in SAS PROC MIXED).

A responder analysis summarised the proportion of patients in each treatment arm who experienced clinically meaningful improvement at ⩾1 post-baseline assessment for each EORTC QLQ-C30 scale (HRQOL, functional, and symptoms). A clinically meaningful improvement was defined as a ≥10-point change from baseline within a patient’s self-rated score (Osoba *et al*, 1998) and conducted for each treatment arm separately.

*Post hoc* analyses in the PRO population evaluated the impact of symptomatic adverse events of special interest (i.e., pyrexia, diarrhoea, photosensitivity, rash, and serous retinopathy) on HRQOL by comparing mean changes from baseline in the GHS score for patients in the treatment arm who experienced each AE (any grade) at any time point during the study and for patients in the treatment arm who did not experience the AE at each time point through cycle 8 day 1.

## Results

### Patient population

Between January 2013 and January 2014, a total of 495 patients were enrolled at 133 sites in 19 countries and randomly assigned to receive first-line treatment with cobimetinib combined with vemurafenib (*n*=247) or placebo and vemurafenib (*n*=248). Baseline demographics and disease characteristics have previously been described and were generally well-balanced across treatment groups (Larkin *et al*, 2014). Briefly, the majority of patients were white (93%), male (58%), had an ECOG PS of 0 (70%), and an M1c disease stage at baseline (60%).

**Figure 1:**
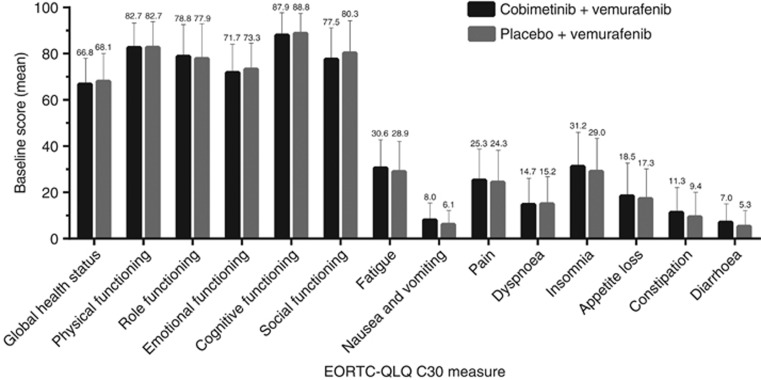
**Mean baseline values for EORTC QLQ-C30.** Error bars are standard error of mean (SEM). High positive scores in the functional domains indicate better functional status, while high positive scores in symptom domains indicate worse symptoms. EORTC QLQ-C30=European Organisation for Research and Cancer Quality of Life Questionnaire Core 30.

On the data cutoff date (9 May 2014), the PRO-evaluable population included 420 patients (211 in the cobimetinib combined with vemurafenib arm and 209 in the placebo and vemurafenib arm), or 85% of the intention-to-treat population. Among the PRO-evaluable population, completion rates of the EORTC QLQ-C30 were 96.7% (202/209) among patients in the placebo and vemurafenib arm and 97.2% (205/211) among patients in the cobimetinib combined with vemurafenib arm at baseline and consistently high (>88%) for all assessments thereafter. Patients in both treatment arms reported moderate-to-high functioning and HRQOL, with minimal symptom burden at baseline (Figure 1). Mean scores for all EORTC QLQ-C30 domains were similar between treatment groups at baseline, with no differences exceeding 5 points.

### Longitudinal analyses

**Figure 2:**
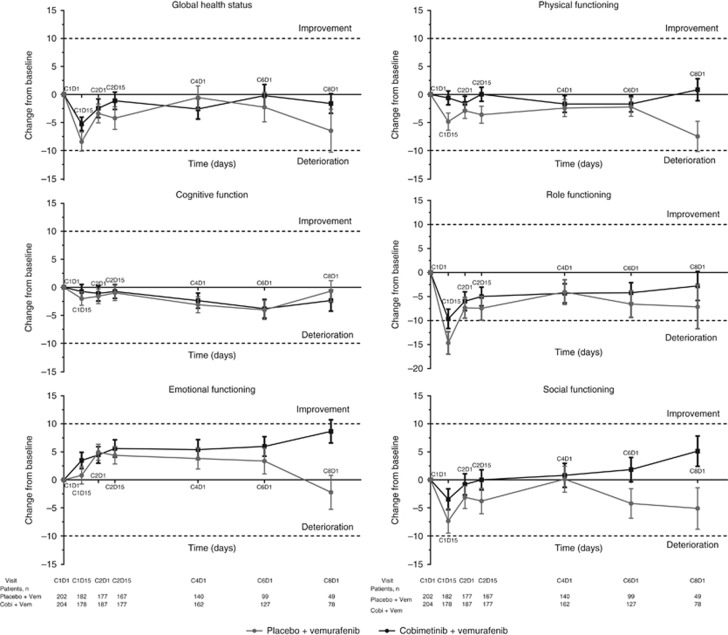
**Mean change from baseline in EORTC QLQ-C30 functional scores at each post-baseline assessment.** Error bars are standard error of mean (SEM). Each treatment cycle was 28 days, with vemurafenib administered on days 1–28 and cobimetinib administered on days 1–21, followed by a 7-day rest period. EORTC QLQ-C30=European Organisation for Research and Cancer Quality of Life Questionnaire Core 30.

**Figure 3:**
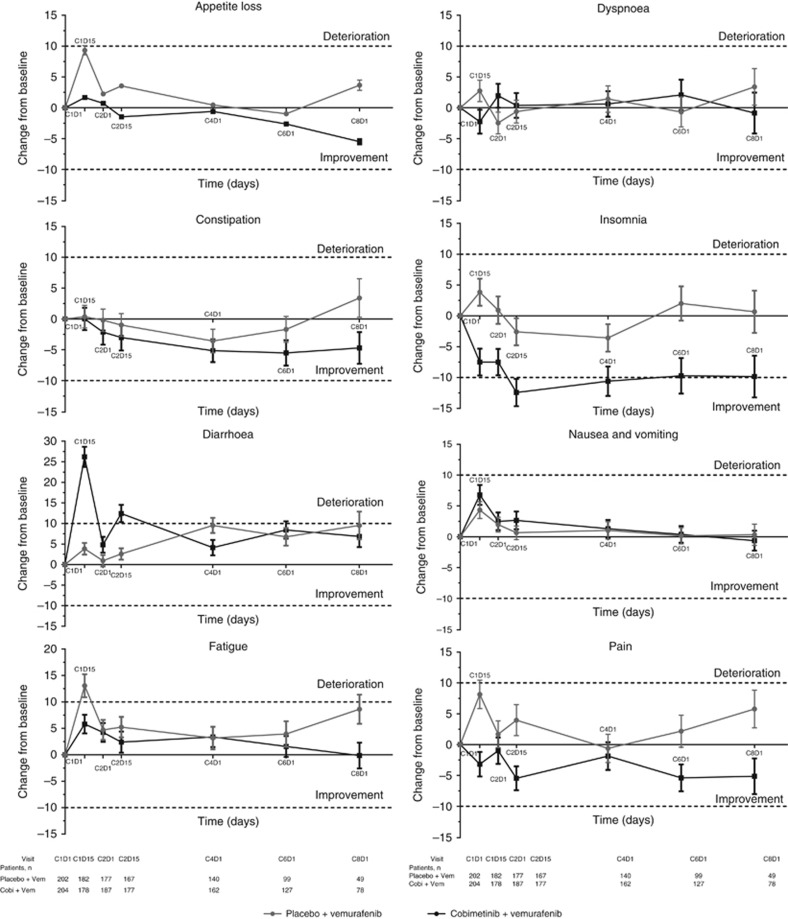
**Mean change from baseline in EORTC QLQ-C30 symptom scores at each post-baseline assessment.** Error bars are standard error of mean (SEM). Each treatment cycle was 28 days, with vemurafenib administered on days 1–28 and cobimetinib administered on days 1–21, followed by a 7-day rest period. EORTC QLQ-C30=European Organisation for Research and Cancer Quality of Life Questionnaire Core 30.

All function domains (Figure 2), most patient-reported symptoms (Figure 3), and HRQOL, as assessed by the EORTC QLQ-C30, were comparable between the two arms. Among the function domains, the only clinically meaningful change from baseline (⩾10 points) was observed in the Role Function domain among patients receiving placebo and vemurafenib at cycle 1 day 15 (mean change from baseline, -14.7 points; Figure 2). By cycle 8 day 1, there was a trend toward improvement for the Emotional, Physical, and Social domains in patients who received cobimetinib combined with vemurafenib and a trend toward deterioration in patients who received placebo and vemurafenib; however, they did not constitute a clinically meaningful change (i.e., change from baseline of ⩽10 points; Figure 2).

Among the symptom domains, a clinically meaningful improvement in Insomnia was observed in patients who received cobimetinib combined with vemurafenib at cycle 2 day 15 (mean change from baseline, -12.4 points; Figure 3) and at cycle 4 day 1 (mean change from baseline, -10.6 points). Clinically meaningful deterioration of diarrhoea symptoms were observed in patients who received cobimetinib combined with vemurafenib at cycle 1 day 15 (mean change from baseline, 26.2 points, Figure 3) and cycle 2 day 15 (mean change from baseline, 12.4 points). Patients who received placebo and vemurafenib reported clinically meaningful deterioration of fatigue at cycle 1 day 15 (mean change from baseline, 13.1 points; Figure 3).

In the mixed-effect model analysis, there were few clinically meaningful differences (⩾10 points) between the cobimetinib combined with vemurafenib arm and the placebo and vemurafenib arm across all functional domains (Supplementary Tables S1 and S2). Among the symptom scales and items (Supplementary Table S2), there was a clinically meaningful improvement in pain in the cobimetinib combined with vemurafenib arm (difference in mean change from baseline between arms, -10.63 points (95% CI: -15.32 to -5.95; *P*<0.0001 at cycle 1 day 15)); the only item favoring the placebo and vemurafenib arm was diarrhoea (difference in mean change from baseline between the arms, 23.24 points at cycle 1 day 15 (95% CI: 18.87–27.61; *P*<0.0001) and 11.75 points at cycle 2 day 15 (95% CI: 7.29–16.21; *P*<0.0001)).

### Responder analysis

**Figure 4:**
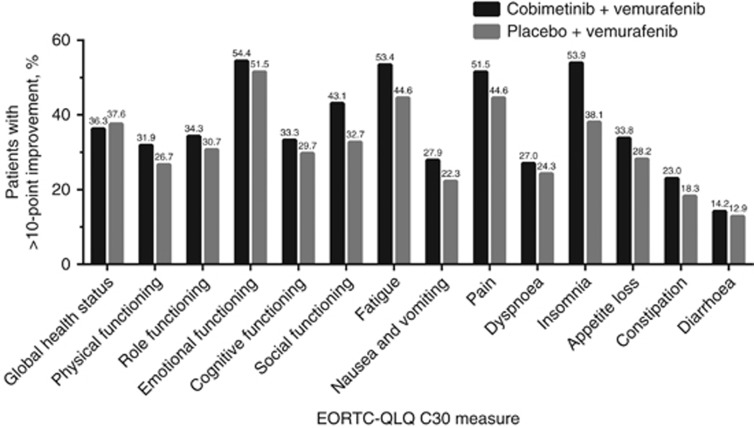
**Proportion of patients with clinically meaningful improvement in EORTC QLQ-C30 scores at ⩾1 post-baseline time point.** Clinically meaningful improvement is defined as a ⩾10-point increase in scores for global health status and functioning scales or a ⩾10-point decrease in scores for symptom scales. EORTC QLQ-C30=European Organisation for Research and Cancer Quality of Life Questionnaire Core 30.

For GHS, as well as most functioning and symptom scales, the difference in the proportion of responders in each treatment arm was approximately <5%, indicating similarity in HRQOL between the two treatment arms (Figure 4). The differences between groups were greatest for insomnia (16%), social functioning (10%), fatigue (9%), and pain (7%), all favouring the cobimetinib combined with vemurafenib arm.

### Impact of select symptomatic AEs on GHS

**Figure 5:**
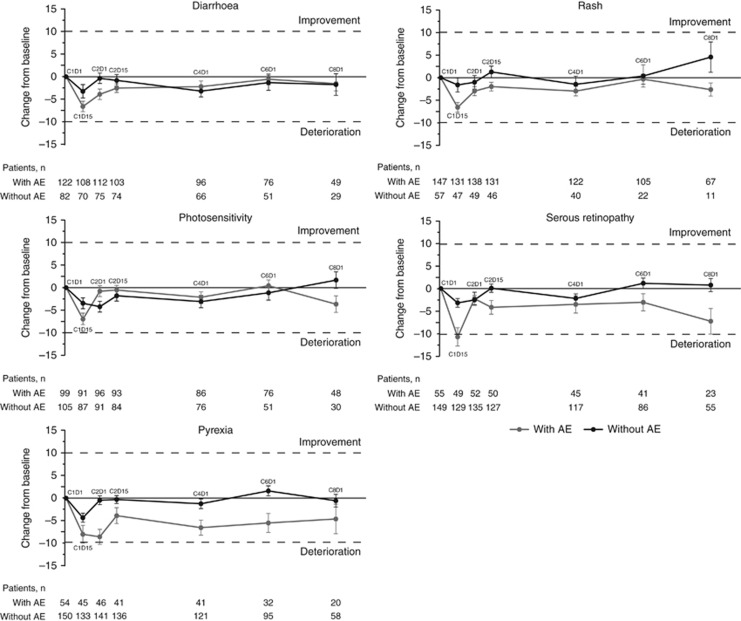
**Impact of select AEs on global health status.** Clinically meaningful change is defined as a ⩾10-point change from baseline. Error bars are standard error of mean (SEM). Each treatment cycle was 28 days, with vemurafenib administered on days 1–28 and cobimetinib administered on days 1–21, followed by a 7-day rest period. AE=adverse event.

Diarrhoea, photosensitivity, rash, pyrexia, and serous retinopathy (any grade) were assessed for impact on patient-reported HRQOL (Figure 5). There was no clinically meaningful deterioration at any time point in HRQOL for four of the five AEs (exception of serous retinopathy), as indicated by the absence of changes in GHS scores ⩾10 points in patients who experienced each AE during the study. Patients who experienced serous retinopathy reported a transient but clinically meaningful decrement in GHS early in treatment (cycle 1 day 15 assessment). However, at all later assessments, GHS was similar to the baseline score in patients who experienced serous retinopathy.

## Discussion

The primary analysis of the coBRIM study demonstrated that the combination of cobimetinib and vemurafenib improved PFS and objective response rates compared with placebo and vemurafenib in patients with advanced or metastatic *BRAF*^V600^ mutation–positive melanoma, with a manageable increase in the incidence of AEs (Larkin *et al*, 2014). Although improved survival is paramount, preserving patient HRQOL is a critically important consideration in real-world practice. This double-blind study that yielded a consistently high completion rate for the EORTC QLQ-C30 provides a robust source of PRO data to better understand treatment and disease burden in patients with melanoma. Overall, the current analyses of the PRO data indicate that cobimetinib combined with vemurafenib improves PFS and overall survival (OS) outcomes without compromising HRQOL. The trend towards patients’ functional deterioration (Emotional, Physical, and Social function scores) by cycle 8 in the placebo and vemurafenib arm is possibly reflective of the cumulative impact of both progressive disease and treatment burden for patients remaining on treatment past the time of median PFS (6.2 months), as compared with patients in the cobimetinib combined with vemurafenib arm who have not yet progressed.

Furthermore, data showed that pyrexia, diarrhoea, photosensitivity, and rash (4 out of the 5 most commonly reported symptomatic AEs associated with cobimetinib) did not impact the patients’ overall quality of life (GHS) in a clinically meaningful manner (⩾10 points) at any time point in the treatment arm. The singular exception was among patients who experienced any-grade serous retinopathy, who reported a clinically meaningful decrement in GHS at the cycle 1 day 15 assessment. However, at all subsequent assessments, GHS scores were similar to their baseline score in patients who experienced serous retinopathy. Of note, the observed worsening in patient-reported diarrhoea severity in the combination arm only was also reflected in the clinician report (on CTCAE) of diarrhoea AE (any grade: 56.7% *vs* 28%, grade ⩾3: 6.3% *vs* 0, for the combination and monotherapy arms, respectively) (Larkin *et al*, 2014). Patient ratings of diarrhoea severity underscore the temporal nature of this AE, characterised by a relatively rapid onset of this AE within the first cycles of treatment (median time to first onset, 0.36 months) and return to baseline values afterwards. This pattern is consistent with safety reporting (i.e., CTCAE gradings), with a decline in diarrhoea AE incidence after cycle 2 (Dréno *et al*, 2016, 2017), and the fact that most severe events (grade⩾3) occurred within the first three cycles.

Temporal analyses of diarrhoea, photosensitivity, and serous retinopathy at the primary data cutoff for the PFS analysis (May 2014), compared with that from an extended follow-up (September 2015), show that the incidence rates of all these AEs decreased with continued treatment (Dréno *et al*, 2016, 2017). This is consistent with the observed early onset of these AEs and subsequent resolution with clinical management thereafter (Dréno *et al*, unpublished data).

The double-blind, randomised, phase III COMBI-d study evaluated the BRAF inhibitor dabrafenib in combination with the MEK inhibitor trametinib (*vs* dabrafenib monotherapy) and also reported the effect of the combination on HRQOL using the EORTC QLQ-30 (Schadendorf *et al*, 2015). The baseline and on-treatment HRQOL and symptom scores seen in this study are similar to those reported with the combination of dabrafenib and trametinib compared with dabrafenib and placebo. In coBRIM, patients self-reported higher levels of physical functioning, role functioning, cognitive functioning, and social functioning at baseline compared with those enrolled in COMBI-d; therefore, it is possible that it would be more challenging to demonstrate clinically meaningful improvement from baseline in these respective domains for patients enrolled in coBRIM.

A limitation of the current study is the use of a generic instrument for the evaluation of HRQOL in patients with cancer (EORTC QLQ-C30), rather than a melanoma-specific instrument. Although the EORTC QLQ-C30 is widely validated for the assessment of HRQOL in patients with cancer (Aaronson *et al*, 1993) and has been used across several trials in patients with melanoma (Grob *et al*, 2014; Schadendorf *et al*, 2014; Grob *et al*, 2015; Schadendorf *et al*, 2015), it may not fully address issues specific to patients with melanoma or symptomatic adverse events associated with therapies other than chemotherapies. An EORTC-based melanoma-specific module was recently developed and consists of 33 scoring items, two single items, and three items associated with clinical trials (EORTC QLQ-MEL38), but it has yet to be validated (Winstanley *et al*, 2015). Future studies using melanoma-specific instruments, and instruments that assess treatment-related symptoms associated with the combination therapy such as photosensitivity and rash, might provide greater insight into the effects of treatments on HRQOL issues relevant to patients with melanoma. Additionally, a limitation in the current study is that patients who discontinue treatment due to an AE are not required to continue to complete PRO assessments. However, patients were required to complete the PRO questionnaires immediately when entering the clinic, prior to the performance of non-PRO assessments (including discussions regarding health status and AEs) and prior to formal treatment discontinuation; therefore, it is reasonable to believe that patients experiencing higher grade AEs would have completed their PRO assessment prior to formal treatment discontinuation and therefore their patient-reported outcome data for that visit would reflect the patient’s AE.

In conclusion, the combination of cobimetinib and vemurafenib provides patient-reported symptoms, function, and HRQOL (with the exception of diarrhoea) similar to that of placebo and vemurafenib. These results, along with the previously reported improved PFS, response rate, and OS (Larkin *et al*, 2014; Atkinson V, 2015), suggest that cobimetinib combined with vemurafenib is a superior treatment for patients with advanced or metastatic *BRAF*^V600^ mutation–positive melanoma compared with vemurafenib alone.
