# Supplementary material for: Health-related quality of life impact of cobimetinib in combination with vemurafenib in patients with advanced or metastatic BRAFV600 mutation–positive melanoma
Source: Br J Cancer. 2018 Feb 13;118(6):777–84. doi: 10.1038/bjc.2017.488 (PMC5877437; doi:10.1038/bjc.2017.488)
Supplement: Supplementary Table 1 [file bjc2017488x1.docx]

| **Supplemental Table 1. Mixed-model difference in change from baseline in EORTC QLQ-C30 functional domains (mean cobimetinib combined with vemurafenib score minus mean placebo and vemurafenib score)^a^** | | | | | | |
| --- | --- | --- | --- | --- | --- | --- |
| **Functional Domain** | **Difference in Mean Change From Baseline Scores for Cobimetinib Combined With Vemurafenib *vs* Placebo and Vemurafenib (95% CI), *P*-value** | | | | | |
|  | **C1D15^b^** | **C2D1^b^** | **C2D15^b^** | **C4D1^b^** | **C6D1^b^** | **C8D1^b^** |
| Global health status | 2.31 (−1.65 to 6.28), 0.25 | 0.31 (−4.26 to 3.65), 0.88 | 2.01 (−2.02 to 6.05), 0.33 | −0.36 (4.60 to 3.88), 0.87 | 1.87 (−2.88 to 6.62), 0.44 | 1.44 (−4.66 to 7.55), 0.64 |
| Physical functioning | 3.96 (0.73 to 7.20), 0.02 | 0.86 (−2.37 to 4.09), 0.60 | 3.50 (0.21 to 6.78), 0.04 | 1.07 (−2.36 to 4.51), 0.54 | −0.56 (−4.36 to 3.24), 0.77 | 5.88 (1.04 to 10.67), 0.02 |
| Role functioning | 5.90 (0.87 to 10.93), 0.02 | 1.21 (−3.81 to 6.23), 0.64 | 2.70 (−2.41 to 7.82), 0.30 | 0.80 (−4.59 to 6.18), 0.77 | 1.93 (−4.10 to 7.96), 0.53 | 2.37 (−5.38 to 10.13), 0.55 |
| Emotional functioning | 1.45 (−2.17 to 5.07), 0.43 | −1.93 (−5.54 to 1.68), 0.29 | −0.37 (−4.05 to 3.31), 0.85 | 1.0 (−2.87 to 4.87), 0.61 | 2.11 (−2.21 to 6.43), 0.34 | 5.57 (0.03 to 11.11), <0.05 |
| Cognitive functioning | 0.89 (−2.36 to 4.14),  0.59 | 0.01 (−3.23 to 3.25), 0.99 | −0.56 (−3.86 to 2.74), 0.74 | −0.40 (−3.86 to 3.06), 0.82 | −0.38 (−4.21 to 3.42), 0.85 | −2.41 (−7.27 to 2.46), 0.33 |
| Social functioning | 3.17 (−1.48 to 7.82), 0.18 | 0.27 (−4.36 to 4.91), 0.91 | 2.92 (−1.80 to 7.65), 0.23 | 0.97 (−3.99 to 5.93), 0.70 | 5.08 (−0.46 to 10.61), 0.07 | 3.03 (−4.05 to 10.11), 0.40 |
| C1D15 = cycle 1 day 15; C2D1 = cycle 2 day 1; C2D15 = cycle 2 day 15; C4D1 = cycle 4 day 1; C6D1 = cycle 6 day 1; C8D1 = cycle 8 day 1; CI = confidence interval; EORTC QLQ-C30 = European Organisation for Research and Treatment of Cancer Quality of Life Questionnaire Core 30.  ^a^For global health status and functioning scales, an increase in scores indicates improvement. Positive values indicate a larger change from baseline for the cobimetinib combined with vemurafenib arm *vs* the placebo and vemurafenib arm.  ^b^Each treatment cycle was 28 days, with vemurafenib administered on days 1-28 and cobimetinib administered on days 1-21, followed by a 7-day rest period. | | | | | | |
