# Supplementary material for: Health-related quality of life impact of cobimetinib in combination with vemurafenib in patients with advanced or metastatic BRAFV600 mutation–positive melanoma
Source: Br J Cancer. 2018 Feb 13;118(6):777–84. doi: 10.1038/bjc.2017.488 (PMC5877437; doi:10.1038/bjc.2017.488)
Supplement: Supplementary Table 2 [file bjc2017488x2.docx]

| **Supplemental Table 2. Mixed-model difference in change from baseline in EORTC QLQ-C30 symptom domains (mean cobimetinib combined with vemurafenib score minus mean placebo and vemurafenib score)^a^** | | | | | | |
| --- | --- | --- | --- | --- | --- | --- |
| **Symptom Domain** | **Difference in Mean Change From Baseline Scores for Cobimetinib Combined With Vemurafenib *vs* Placebo and Vemurafenib (95% CI), *P*-value** | | | | | |
|  | **C1D15^b^** | **C2D1^b^** | **C2D15^b^** | **C4D1^b^** | **C6D1^b^** | **C8D1^b^** |
| Fatigue | −6.58 (−10.94 to −2.22), 0.003 | 0.37 (−3.98 to 4.72), 0.87 | −1.52 (−5.95 to 2.92), 0.50 | 0.30 (−4.37 to 4.97), 0.90 | 0.82 (−6.05 to 4.41), 0.76 | −4.42 (−11.13 to 2.30), 0.20 |
| Nausea and vomiting | 3.85 (0.81 to 6.88), 0.01 | 2.52 (0.50 to 5.54), 0.10 | 3.35 (0.26 to 6.45), 0.03 | 2.05 (−1.24 to 5.33), 0.22 | 1.77 (−1.98 to 5.52), 0.36 | 0.34 (−4.62 to 5.31), 0.89 |
| Pain | −10.63 (−15.32 to −5.95), <0.0001 | −2.05 (−6.73 to 2.62), 0.39 | −9.16 (−13.93 to −4.38), 0.0002 | −1.32 (−6.35 to 3.72), 0.61 | −3.49 (−9.18 to 2.19), 0.23 | −5.28 (−12.67 to 2.11), 0.16 |
| Dyspnoea | −5.23 (−9.59 to −0.87), 0.02 | 4.55 (0.20 to 8.90), 0.04 | 0.58 (−3.85 to 5.02), 0.80 | −0.11 (−4.78 to 4.56), 0.96 | 4.10 (−1.13 to 9.33), 0.12 | 0.96 (−5.77 to 7.69), 0.78 |
| Insomnia | −9.98 (−14.9 to −5.01), <0.0001 | −6.27 (−11.22 to −1.31), 0.01 | −9.02 (−14.07 to −3.96), 0.0005^b^ | −5.41 (−10.73 to −0.09), < 0.05 | −6.10 (−12.08 to −0.12), <0.05 | −4.40 (−12.10 to 3.31), 0.26 |
| Appetite loss | −6.49 (−11.95 to −1.02), 0.02 | −0.80 (−6.24 to 4.65), 0.77 | −4.51 (−10.08 to 1.05), 0.11 | −0.29 (−6.18 to 5.59), 0.92 | −2.05 (−8.71 to 4.61), 0.55 | −4.05 (−12.75 to 4.64), 0.36 |
| Constipation | 0.65 (−3.01 to 4.32), 0.73 | −0.57 (−4.22 to 3.08), 0.76 | 0.47 (−3.27 to 4.21), 0.81 | 0.63 (−3.33 to 4.59), 0.76 | −0.86 (−5.36 to 3.65), 0.71 | −6.28 (−12.22 to −0.35), 0.04 |
| Diarrhoea | 23.24 (18.87 to 27.61), <0.0001 | 5.00 (0.65 to 9.36), 0.02 | 11.75 (7.29 to 16.21), <0.0001 | −2.80 (−7.53 to 1.94), 0.25 | 3.33 (−2.07 to 8.72), 0.23 | −1.84 (−8.98 to 5.30), 0.61 |
| Shading represents clinically meaningful differences (≥10 points) between the cobimetinib combined with vemurafenib arm and placebo and vemurafenib arm.  C1D15, cycle 1 day 15; C2D1, cycle 2 day 1; C2D15, cycle 2 day 15; C4D1, cycle 4 day 1; C6D1, cycle 6 day 1; C8D1, cycle 8 day 1; CI, confidence interval; EORTC QLQ-C30, European Organisation for Research and Treatment of Cancer Quality of Life Questionnaire.  ^a^For symptom scales, a decrease in scores indicates improvement. Negative values indicate a larger change from baseline for the cobimetinib combined with vemurafenib arm *vs* the placebo and vemurafenib arm.  ^b^Each treatment cycle was 28 days, with vemurafenib administered on days 1-28 and cobimetinib administered on days 1-21, followed by a 7-day rest period. | | | | | | |
